# Characterization of *Streptococcus pyogenes* Strains from Tonsillopharyngitis and Scarlet Fever Resurgence, 2023—FIRST Detection of M1_UK_ in Bulgaria

**DOI:** 10.3390/microorganisms13010179

**Published:** 2025-01-16

**Authors:** Emma Keuleyan, Theodor Todorov, Deyan Donchev, Ani Kevorkyan, Radoslava Vazharova, Alexander Kukov, Georgi Todorov, Boriana Georgieva, Iskra Altankova, Yordanka Uzunova

**Affiliations:** 1 Clinical Microbiology and Virology Laboratory, University Hospital “Lozenetz”, 1407 Sofia, Bulgaria; georgi060606@gmail.com; 2 Laboratory of Medical Genetics and Molecular Biology, University Hospital “Lozenetz”, 1407 Sofia, Bulgaria; theodor_todorov@hotmail.com (T.T.); radvazh@abv.bg (R.V.); 3 Medical Faculty, Sofia University “St. Kliment Ohridski”, 1407 Sofia, Bulgaria; 4 National Reference Laboratory for Control and Monitoring of Antimicrobial Resistance, National Center for Infectious and Parasitic Diseases, 1504 Sofia, Bulgaria; deyandonchev@ncipd.org; 5 Department of Epidemiology and Disaster Medicine, Faculty of Public Health, Medical University—Plovdiv, 4002 Plovdiv, Bulgaria; ani_kevorkyan@mu.plovdiv; 6 Laboratory of Clinical Immunology, University Hospital “Lozenetz”, 1407 Sofia, Bulgaria; a_dimitroff@abv.bg (A.K.); altankova@abv.bg (I.A.); 7 Clinic of Pediatrics, University Hospital “Lozenetz”, 1407 Sofia, Bulgaria; borianaemgeorgieva@gmail.com (B.G.); daniuz@gbg.bg (Y.U.)

**Keywords:** *Streptococcus pyogenes*, virulence factors, antibiotic susceptibility, M1_UK_, tonsillopharyngitis, scarlet fever, immune responses

## Abstract

Recently a resurgence of *Streptococcus pyogenes infections* has arisen, with concerns around the highly virulent M1_UK_ lineage. Our aim was to characterize *S. pyogenes*, the immune responses it causes, and to determine the presence of the M1_UK_ lineage in Sofia, Bulgaria. In our study, the infections were confirmed by culture testing or rapid antigen test. Identification was performed by MALDI-TOF and was followed up by antibiotic susceptibility testing (EUCAST). Virulence factors were identified using multiplex PCR and whole genome sequencing (WGS). Immune responses were measured through detection of serum complement levels, lymphocyte subsets, and cytokine profiling. Out of 82 children, 38 had scarlet fever and the rest had streptococcal pharyngitis. Strains were susceptible to penicillin (β-lactams), macrolides, clindamycin, tetracyclines, co-trimoxazole, fluoroquinolones, and linezolid. Superantigen profiles were identified: SpeA + SpeJ (45%), SpeC, and SpeI + SpeH (27.5% each). A novel Multilocus sequence typing (MLST) haplotype in the *mut*S gene (d90b) was found in four strains. The M1_UK_ lineage was detected for the first time in Bulgaria. We observed an increase in complement fractions C3 and C4 and a decrease in T lymphocytes. A significant increase in the levels of IFN-γ, IL-6, and IL-10 with corresponding reduction in IL-17A were revealed. In conclusion, the studied *S. pyogenes* strains were characterized by their susceptibility to antibiotics and the predominance of SpeA superantigen; for the first time in Bulgaria the presence of M1_UK_ and a novel SNP variation in the *mut*S gene (d90b) were found. A mixed pattern of pro- and anti-inflammatory immune responses in patients was observed.

## 1. Introduction

*Streptococcus pyogenes* or Group A beta-haemolytic *Streptococcus* (GAS) is a well known Gram-positive, human pathogen causing a persistent, global burden of disease, which again attracts attention of the medical specialists [1,2,3]. Of particular interest is the variety of produced virulence factors [4,5,6,7,8] and induced infections [9,10]. It should be mentioned that this microorganism is one of the most frequent bacterial pathogens causing tonsillopharyngitis (12 million health related visits in the USA alone, the majority of them being viral) [11]. Moreover, in case of a non-diagnosed GAS tonsillopharyngitis, suppurative complications can occur, such as otitis media, sinusitis, and peritonsillar abscess. Another group of complications connected with GAS are the immune-related ones, such as acute rheumatic fever (ARF) and post-streptococcal glomerulonephritis [12,13,14]. Although auto-immune sequelae significantly diminished in the past two decades, ARF continues to cause morbidity and mortality in some low/middle income countries and in certain geographic regions. In 2021 there were approximately 373,000 deaths caused by rheumatic heart disease (RHD) and 13.4 million disability adjusted life-years (Global Burden of Diseases) [15]. Scarlet fever is another frequent presentation of GAS infection in children with epidemic distribution and seasonal presentation typically during spring [16,17]. Two other GAS-associated diseases—erysipelas and impetigo, affect predominantly adults and are generally encountered in the warm summer months. *S. pyogenes* strains could also cause severe invasive diseases via septic distribution, with necrotizing fasciitis and streptococcal toxic shock syndrome (STSS) as the most dangerous, due to the extensive production of exotoxins [18].

The majority of contemporary investigations on *S. pyogenes* are focused on epidemiology of the infections: distribution and dynamics of *emm* types, representing the main *S. pyogenes* antigen—M protein, which has more than 261 types. This diversity makes global comparisons of strains complex [19], as well as making a successful vaccine for prevention difficult. The World Health Organization (WHO) determines *S. pyogenes* among its top priority microorganisms, as it causes frequent endemic outbreaks, and effective vaccines should be developed as soon as possible [20].

It has been shown that *S. pyogenes* has a plethora of virulence factors (M proteins, hyaluronic acid capsule, superantigens, etc.) that could induce a strong early immune response of the host innate and adaptive immunity [1,3,5]. Mucosal-associated invariant T cells (MAIT) and γδTCR + Vδ2 + T cells play an essential role in the mucosal response against GAS infection via the secretion of significant amounts of IFN-γ, TNFα, and IL-2. These cytokines prepare and organize the subsequent specific anti-GAS immune response [5,6]. In acquired immunity, an important role is played through Th1, Th17 sub cells, and the cytokines produced by them (IL-6, TNFα, IFN-γ, etc.) [1,3,7]. Nevertheless, a better understanding of the effective host immunological responses is needed. Therefore, we studied some humoral and cellular immune parameters during an infection with *S. pyogenes* presenting as tonsillopharyngitis or scarlet fever in children.

In recent years, two important events, regarding *S. pyogenes*, arose. The first of them is the emergence of a new virulent M1_UK_ lineage [21]. It differs from the widely distributed M1_global_ by increased expression (9.5 time more) of the exotoxin superantigen SpeA, due to changes in 27 single nucleotide polymorphisms (SPNs). The M1_UK_ was first associated with increased incidence of scarlet fever and invasive infections in England during the period 2015–2016 [21]. Demonstrating remarkable finesse in pathogenicity, M1_UK_ became predominant in the country and spread to Australia [22], Canada [23], and the USA [24] and later in some European countries (Netherlands [25], Denmark [26], Ireland and Scotland [27], Belgium [28], and Portugal [29] and then in Italy [30], Spain [31], Germany [32], and others). This lineage is strongly associated with increased morbidity and mortality of severe invasive infections, requiring intensive care admission (sepsis, pneumonia, pleural empyema, tissue infections, STSS, etc.) [27,28,29,30,31,32]. While the M1_UK_ lineage has been documented across Europe, its presence in Eastern Europe with its associated immunological response remains understudied.

Another important event was the increase in cases of both streptococcal tonsillopharyngitis and scarlet fever, as well as invasive GAS infections in 2022–2023, announced by the CDC, Atlanta, USA, ECDC, and WHO [33,34,35]. Scientists from North, West, and Central European countries described a significant increase in GAS diseases, including severe cases [25,26,27,28,29,30,31,32,36,37]. In Bulgaria, an increase in cases of GAS tonsillopharyngitis and scarlet fever started in the beginning of 2023.

Our prospective study was conducted on children infected with GAS (both hospitalized and outpatients) in the Pediatric Department of University Hospital “Lozenetz”, Sofia between 1 January and 30 June 2023. The study included patients diagnosed with streptococcal tonsillopharyngitis and scarlet fever. The primary aim was to characterize the isolated *S. pyogenes* strains, focusing on their antibiotic susceptibility, key virulence factors, and specific genetic characteristics. Additionally, the study aimed to assess the immune response triggered in the infected children. Furthermore, a critical objective was to determine whether the M1_UK_ lineage was present in Bulgaria.

## 2. Materials and Methods

### 2.1. Study Setting, Participants, and Sample Collection

The design of this prospective study included screening of 82 GAS-infected children (both inpatients and outpatients) at the Pediatric Department of University Hospital “Lozenetz”, Sofia, Bulgaria between 1 January and 30 June 2023 with either streptococcal tonsillopharyngitis or scarlet fever. Informed consent was signed by the children’s parents and guardians. The studied children were tested by microbial culture and/or rapid OSOM Strep A Test (SEKISUI Diagnostics, Burlington, MA, USA). For the immunological study, 22 children with proven GAS infection and clinical manifestation of tonsillopharyngitis (18) and scarlet fever (4) were included. The mean age was 7 ± 1.84 years; ten of the patients were girls and twelve were boys. Two control groups of ten children each were included in the study. Control group 1 (used for comparison of humoral and cellular immunity) consisted of ten children: eight boys and two girls; mean age 5.9 ± 2.7 years. Control group 2 consisted of ten children: six boys and four girls; mean age 10.9 ± 3.04 years, which was used for comparison of cytokine parameters between sick patients and healthy individuals. The blood samples for the immunological tests were taken at the moment of the inclusion of patients in the study.

### 2.2. Routine Microbiological Culture Method

The collected nasopharyngeal swabs were cultured on Columbia blood agar and incubated in 5% CO_2_ at 35 °C for 24–48 h. Identification of the suspected colonies was performed by MALDI-TOF (Bruker, Bremen, Germany).

### 2.3. Antimicrobial Susceptibility Testing

Antimicrobial susceptibility testing (AST) of the isolates towards clinically used antimicrobial agents (penicillin, erythromycin, clindamycin, tetracycline, levofloxacin, moxifloxacin, linezolid, and co-trimoxazole) was evaluated on Mueller–Hinton agar with 5% horse blood and 20 mg/L nicotine amide dinucleotide (NAD) (Graso Biotech, Gdansk, Poland) by the disc diffusion method, according to the EUCAST, 2024. Minimal Inhibitory Concentration (MIC) to several antibiotics for 30 randomly selected strains was determined via MIC test strips (E-tests and gradient strip method). The bacterial inoculum of 0.5 MacFarland (~1.5 × 10^8^ CFU/mL) prepared from 4 to 6 colonies in saline solution was struck by swab 3 times, changing the position by 60° at the Muller–Hinton agar with horse blood and NAD. On the surface of the Petri dishes (9 cm diameter), two E-test strips (BioMérieux, Marcy-l’Étoile, France) were placed opposite to each other and cultivated for 18 h at 35 °C in 5% CO_2_. The MIC value reading was according to the position where the ellipse zone of growth inhibition crossed the graduated scale of the E-test.

### 2.4. Molecular Detection of Virulence Factors and M1_UK_

Virulence genes were detected using multiplex PCR. Primers to the virulence genes (*spe*A, *spe*C, etc.) were used as previously described by Gammoh NZ et al. [38] (Supplementary Table S1). The PCRs were carried out in a 25 μL volume containing 2.5 μL of 10× PCR buffer, 2.5 mM MgCl_2_, 200 μM dNTPs, 0.2 μM of each primer, 1 U Taq DNA polymerase (Taq DNA Polymerase, Roche, Basel, Switzerland), and 50 ng of genomic DNA. The PCR conditions were as follows: initial denaturation at 95 °C for 5 min, followed by 35 cycles of 95 °C for 30 s, annealing at the appropriate temperature for each primer pair for 30 s, and extension at 72 °C for 1 min, with a final extension at 72 °C for 7 min. The PCR products were analyzed by gel electrophoresis on a 1.5% agarose gel. To distinguish between the M1_global_ and M1_UK_ lineages of *Streptococcus pyogenes*, additional allele-specific PCR primers were used as described by Zhi X et al. [39], as shown in Table 1. The PCR conditions for these primers were the same as those described above, with specific annealing temperatures optimized for the differentiation between the M1_global_ and M1_UK_ lineages.

### 2.5. Whole Genome Sequencing (WGS)

Whole genome sequencing was conducted by randomly fragmenting the genomic DNA using Covaris. Libraries were prepared using the BGISEQ-500 platform (BGI Tech Solutions, Hong Kong, China) with 2 × 150 bp PE reads. See Supplementary Tables S2–S4 for further details. Raw sequencing data were processed using FastQC v0.12.0 for quality control, and reads were trimmed using Trimmomatic v0.39. Genome assembly was performed using Unicycler v0.5.0 with default parameters. Contigs shorter than 200 bp were removed. The assembled genomes were annotated automatically using PGAP upon upload to the NCBI Genbank. The presence of the M1_UK_ variant was confirmed by aligning the sequences to the reference genome NCTC12064 using BWA-MEM v0.7.17 (r1188), followed by variant calling with FreeBayes v1.3.7. Genomes were checked with CheckM v1.2.2. and Kmerfinder v3.0.2 with database (DB) v2022-07-11 for contamination and completeness. Species identification was performed with GTDBTK v 2.3.2.

### 2.6. Bioinformatic Analyses

*emm* genotyping and Multi-locus Sequence Typing (MLST) were performed using the assembled genomes with the public PubMLST *emm* typing scheme and the 7-loci MLST *S. pyogenes* scheme (https://pubmlst.org/, accessed on 8 May 2024), respectively. Virulence determinants were identified with AMRFinderPlus v3.11.4 [40] and analyzed with DB v2023-01-18. Virulence genes were extracted with VFanalyzer (http://www.mgc.ac.cn/cgi-bin/VFs/v5/main.cgi?func=VFanalyzer, accessed on 8 May 2024).

### 2.7. Immunological Methods

Total serum immunoglobulins G, M, and A (IgM, IgM, IgA), as well as C3 and C4 complement levels, were measured by appropriate reagent kits (Abbott, Chicago, IL, USA), performed on Alinity automated analyzer (Abbott, Chicago, IL, USA).

Peripheral blood lymphocyte subsets: CD3^+^ T cells, CD4^+^ T helper cells, CD8^+^ cytotoxic T cells, B cells, and NK cells were determined using BD Multitest 6-colour TBNK kit (BD Biosciences, Franklin Lakes, NJ, USA), according to the manufacturer’s instructions. At the beginning of the analysis, 50 μL of whole blood was stained with 20 μL of a 6-colour TBNK antibody cocktail for 20 min. After adding 450 μL of 1× BD FACS Lysing Solution (BD Biosciences) and after 15 min of incubation, the samples were analyzed using FACS Canto II flow cytometer (BD). All data were analyzed using BD FACS Canto v3.0 software. The percentages of lymphocytes were obtained.

Serum levels of cytokines IFN-γ, IL-6, IL-1β, TNFα, IL-17A, and IL-10 were measured with appropriate ELISA kits (Diaclone SAS, Besancon, France), according to the manufacturer’s instructions.

### 2.8. Statistical Analysis

The Kolmogorov–Smirnov test was used to determine the distribution of raw data, and Student’s *t* test and the Mann–Whitney U test were used for comparisons of the data. Differences at *p* < 0.05 were considered significant. Statistical software IBM SPSS Statistics 25 and GraphPad Prism 10 were used.

## 3. Results

### 3.1. Brief Epidemiology

**Figure 1:**
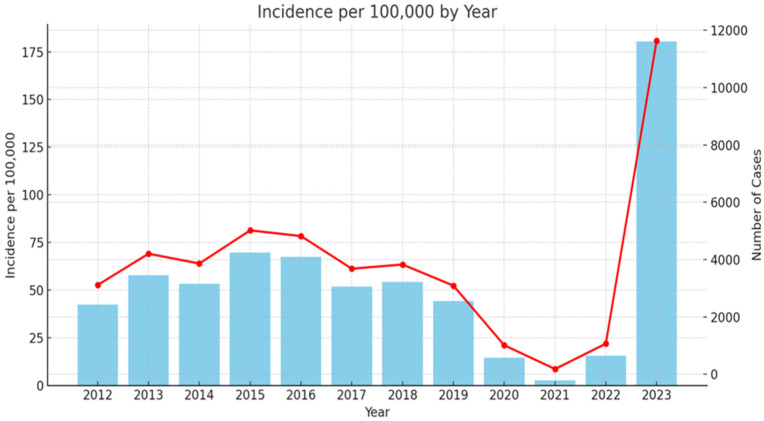
Epidemiology rate of scarlet fever in Bulgaria 2012–2023. Red line—incidence of scarlet fever cases per 100,000 inhabitants per year (left *y*-axis). Blue columns—number of scarlet fever cases per year (right *y*-axis).

Scarlet fever is a widespread infection in Bulgaria since 1897 [41]. During the last decades, similar to other European countries, there were low levels of infection. As it can be seen from Figure 1, in 2023, the country experienced a significant resurgence of cases: 11,634, with incidence of 180.44 per 100,000 people (the Scarlet fever morbidity data collection was based on official annual country information for 2012–2023, extracted from National Centre of infectious and parasitic diseases (NCIPD) (https://ncipd.org/index.php?option=com_k2&view=item&layout=item&id=84&Itemid=1337&lang=bg, accessed on 8 May 2024)).

### 3.2. Clinical Characterization of Children Infected with GAS in Pediatric Department

During the six-month period, the included 82 patients experienced common symptoms of tonsillopharyngitis: sore throat, difficulty swallowing, high fever, and malaise. The clinical manifestation was mainly characterized by enlarged and hyperemic tonsils with purulent exudates and soft palate petechiae. GAS strains were isolated from 50 children (61%), and in the rest of the patients (32) the infection was confirmed by a rapid Strep test. Patients with scarlet fever (38 of 82) also had the typical skin rash. The most prescribed antibiotic was Amoxicillin/clavulanic acid as a 10-day regimen. None of the children experienced early or late complications.

### 3.3. Microbiological Characterization of GAS

#### 3.3.1. Antimicrobial Susceptibility Testing (AST)

According to AST, the strains were susceptible, as expected, to penicillin (first choice antibiotic), erythromycin (second choice), and to other antimicrobial agents used to treat GAS infection of different sites: clindamycin, tetracycline, moxifloxacin, linezolid, and co-trimoxazole. Susceptibility to levofloxacin was intermediate (requiring higher dosage). More detailed information about the antimicrobial susceptibility, namely MIC of five clinically used antimicrobial agents towards 30 randomly selected strains, is presented on Table 2.

These results confirmed the data obtained by the disc diffusion method. As it can be seen from Table 2, all strains were highly susceptible to antimicrobial agents, except the slightly increased MIC of levofloxacin.

#### 3.3.2. GAS Virulence Factors

A variety of virulence factors were detected in the studied strains. We conducted screening of the key prophage-determined exotoxin superantigen genes in a selected group of 40 strains, using multiplex PCR (Table 3).

Among the most important virulence factors in scarlet fever and other GAS-related diseases are the *Streptococcus* pyrogenic exotoxins, which include the superantigens SpeA, SpeB (a chromosome-encoded protease), SpeC, and others. Additionally, sdaD is a DNA-degrading enzyme, while streptodornase (spd3) functions as a deoxyribonuclease. The majority of tested strains (45%) produce the pyrogenic exotoxin SpeA in combination with SpeJ, as well as deoxyribonucleases. Two additional groups of strains produce either SpeC or a combination of SpeI and SpeH, with each group accounting for 27.5% of the strains.

### 3.4. Whole Genome Sequencing of GAS Strains

**Figure 2:**
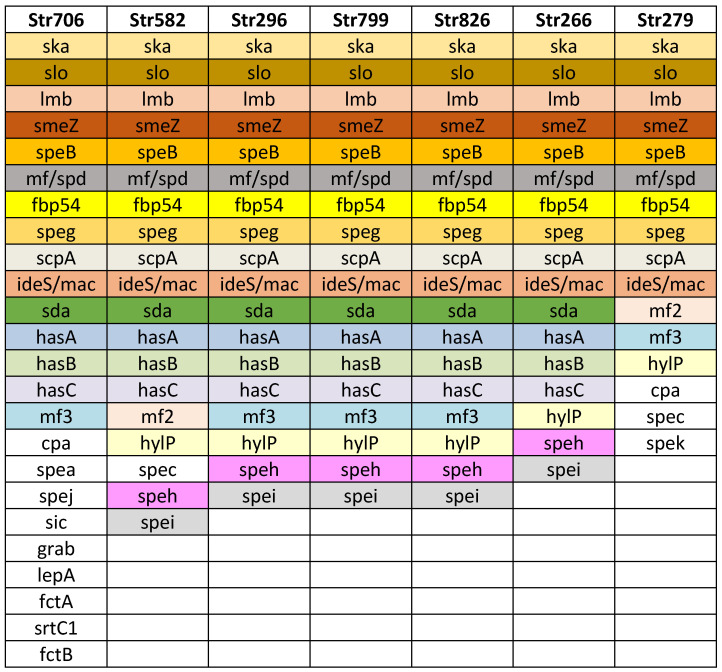
Virulence factor genes identified in the seven *Streptococcus pyogenes* genomes. Strain names are listed in the first row, in columns are listed detected virulence genes. The strain Str 706 was found to belong to the M1_UK_ lineage.

Next, we performed WGS on seven *S. pyogenes* strains obtained from infected children. These strains were selected based on their *spe* superantigen genes (see Section 3.3.2). The WGS analysis allowed the identification of various virulence factors and offered deeper insight of the genetic diversity, pathogenicity, and adaptability of the strains. This sequencing effort contributed to a more comprehensive understanding of the circulating GAS in Bulgaria (Figure 2).

Additional virulence factors were detected: exotoxin superantigens SpeB, SpeG, SpeK, and SpeZ; Slo (oxygen labile haemolysin); ska, streptokinase (activating plasminogen); encoding DNase: mf/spd, sda; protein mitogen factors mf2, mf3; hasA, hasB, and hasC (hyaluronate synthase, participated in hyaluronic acid capsule); hylP (hyaluronate-lyase, degrading capsule, facilitating lysogenia); participating in adhesion: fibronectin binding protein fbp 54, lepA, fctA, fctB, and srtC1; grab (protein, regulating proteolysis at the bacterial cell surface); protecting factors: sic (protease, streptococcal inhibitor of complement); and ideS/mac (immunoglobulin G degrading enzyme of *S. pyogenes*).

#### 3.4.1. Multilocus Sequence Typing (MLST) Findings

Multilocus Sequence Typing (MLST) was performed on the seven genome assemblies. In four of them, a novel, unique for Bulgaria strain—ST was identified, which differed by one locus (*mut*S). The new *mut*S allele variant consisted of 10 SNPs and is noted as d90b in PubMLST, which has not been previously documented (Supplementary Tables S5 and S6). The remaining three genomes were ST 28, 101, and 242. The data have been deposited with the accession number PRJNA1119284 in the NCBI BioProject database (https://www.ncbi.nlm.nih.gov/bioproject/, accessed on 8 May 2024).

#### 3.4.2. *emm* Genotyping

M-protein types were analyzed in the seven studied strains by WGS. One of the genotyped strains belonged to M1_UK_ lineage, one—to *emm* 89, one—*emm* 12.101, and the remaining four—to *emm* 12 (See Supplementary Table S7).

### 3.5. Detection of M1_UK_ Variant

For the first time, the M1_UK_ variant of *Streptococcus pyogenes* was detected in Bulgaria. Out of the samples screened using allele-specific PCR, two strains tested positive for the M1_UK_ variant. After WGS was performed on one of these M1_UK_ positive strains, there was a confirmation of the presence of the M1_UK_ variant by revealing 27 M1_UK_-specific SNPs (Table 4). On Figure 2 the virulence factors detected in the M1_UK_ variant strain Str 706 are presented. In comparison to the other sequenced strains, it possesses more virulence conferring genes relating to adhesion and protection (Figure 2).

### 3.6. Host Immune Responses

**Figure 3:**
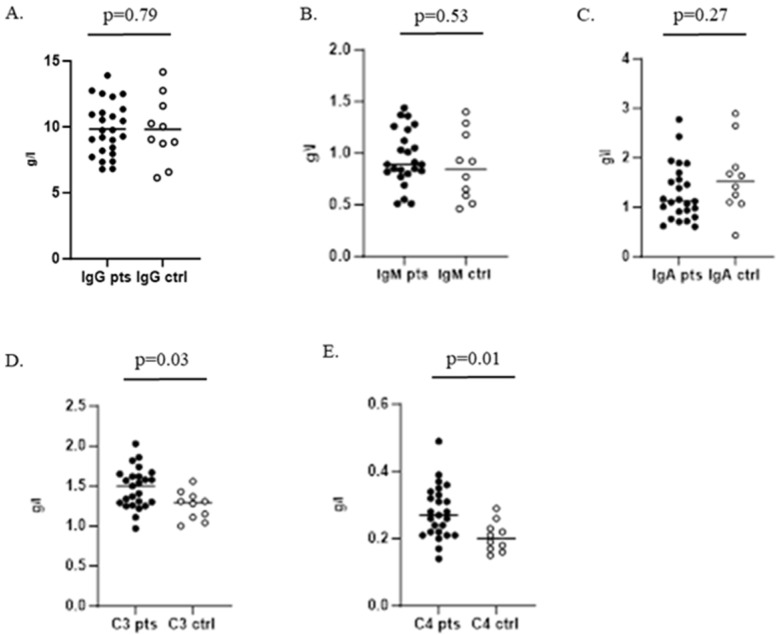
Serum levels of IgG, IgM, and IgA and C3 and C4 complement components in children with GAS infection and healthy controls. (**A**–**C**) Determination of IgG, IgM, and IgA, respectively; (**D**,**E**) represent serum levels of C3 and C4 complement components.

When testing humoral immunity of the infected children, we found no significant difference between serum levels of IgG, IgM, and IgA compared to control group 1. Complement components C3 and C4 were significantly increased in the patients group (Figure 3).

Furthermore, we observed a significant lymphopenia in the GAS infected children (*p* = 0.003). As shown in Table 5, the percentage of CD3^+^ and T helper lymphocytes (CD4^+^) were also significantly decreased in patients compared to controls. Also, we observed a tendency of increased NK cells and no changes in CD8^+^ and B cells (Table 5).

Next, we measured serum levels of IFN-γ, IL-6, TNFα, IL-1β, IL-17A, and IL-10, which might be associated with activation of innate immunity (MAIT and γδT cells) and also with T lymphocytes—adaptive immune responses. We found a significant increase in IFN-γ, IL-6, and IL-10 and reduced levels of IL-17A and no differences in TNFα and IL-1β in patients compared to controls (Table 5).

## 4. Discussion

The sharp increase in infection rates caused by GAS has already been explained by many scientists through a connection with a weakened immunity after the COVID-19 pandemic and the naturally occurring temporal and geographic changes in *S. pyogenes*, which is related to the changes in their virulence and ability to survive [21].

Based on the clinical symptoms and the absence of complications, we concluded that the tonsillopharyngitis and scarlet fever we diagnosed are mild to moderate. A very important aspect for successful therapy is the selection of an appropriate antibiotic. Attempts were conducted to follow the principles of antimicrobial stewardship. The first-choice treatment—penicillin (orally), however, is currently not available in Bulgaria. That is why the majority of children were treated with amoxicillin-clavulanic acid (amoxicillin alone was not available, either). In case of a penicillin allergy, a therapeutic course with a macrolide (clarythromycin) or sometimes a second-generation cephalosporin (cefuroxime) was conducted.

In this study, SpeA was the most frequently found superantigen (45%), as shown in Table 3. The production of SpeA by the majority of invasive and non-invasive strains of *S. pyogenes* has been noted as typical in many European countries [21,26,32]. Moreover, in a recent outbreak in Hong Kong and China, a prevalence of SpeC-producing strains belonging to the M12 serotype was observed [43], with similar findings reported across various European countries [19]. A previous Bulgarian study conducted eight years ago identified Spe exotoxins distribution in different rates: SpeA in 31.5%, SpeC in 28.6%, SpeI in 25.2%, and SpeH in 4.3% of 148 studied strains [44]. This suggests that more studies from other centres are required to truthfully understand the present characteristics of the GAS strains. The additional virulence factors (Figure 2), detected through WGS, once again show *S. pyogenes* as one of the most well-armed bacterial pathogens, allowing adherence and invasion of human tissues by multiple ways, as well as mechanisms of protection from immune reactions.

Another valuable finding was the discovery of a new ST, which differed from ST36 by its *mut*S locus (new allele variant temporarily d90b). This new ST may be important to look into more details as it also circulates among the Bulgarian population. Given that the *mut*S gene plays a critical role in DNA repair, changes in this gene could affect the fidelity of DNA replication and repair mechanisms. Further functional studies are needed to determine the impact of the d90b variant on bacterial fitness and pathogenicity.

We found the M1_UK_ variant for the first time in Bulgaria in two children (Supplementary Table S8). The first child with M1_UK_ *S. pyogenes* was detected on 13 March 2023. She was a 6-year-old preschooler with tonsillopharyngitis. The second one was 7-year-old boy attending elementary school who had three infective episodes of tonsillopharyngitis, each caused by different *S. pyogenes* strains (producing different virulence factors: firstly, SpeC, secondly SpeI + SpeH, and finally SpeA; M1_UK_, detected on 24 June 2023). Both children with M1_UK_ strains had never met each other. As both children and their relatives neither travelled to countries with documented M1_UK_ lineage, nor anywhere abroad, it seems that they have acquired the strains from other previously infected children in preschool and school, respectively (air-drop mechanism of transmission). This suggests that the M1_UK_ lineage had possibly been established in Bulgaria for some time and circulates in the population. It is necessary that more studies from different centres and different regions of the country should be implemented to reveal this spread.

Next, in testing M-protein types by WGS, the majority of tested strains belonged to *emm* 12 (4/7), similarly to the recent Asian outbreak [43], as well as documented in many European countries [45], e.g., in England [46], Portugal [29], Spain [47], Norway [48], Germany [49], usually second rate to M1 _UK_. In Denmark, after the COVID-19 pandemic, during 2022–2023, a 9.5-fold increase in *emm* 12 and a 2.7-fold increase in *emm* 1 was registered [50]. The rate of the GAS *emm* 12 type in previous Bulgarian studies was significantly lower: 11% among strains collected from 2014 to 2017 [44] and 20% in erythromycin resistant isolates collected from 1995 to 2001 [51]. The prevalence of different *emm* types is important for both epidemiology and future vaccines. It has been well documented that *emm* 1 type is the most prevalent in Europe, USA, and Canada [52]. Other frequent types encountered in Europe and North America—at about 60%, are: *emm* 28, *emm* 89, *emm* 3, *emm* 12, *emm* 4, and *emm* 6 [52]. The diversity of *emm* types is higher in low-income countries, and they should also be covered by a potential vaccine. New studies should be performed to better know the current epidemiology of GAS in Bulgaria. This is highly recommended due to the promising perspective of vaccine development, based on M-protein. It should be protective against at least 15 *emm* relevant clusters to be able to sufficiently protect against GAS [19].

Our study of humoral and cellular immunity in children with streptococcal infection showed a typical pattern of host immune responses against microbial infections [53]. We found an increase in serum C3 and C4 components of the complement system, which probably could influence the opsonization of the streptococcal microorganism with subsequent phagocytic destruction, although GAS phagocytosis is limited. In cellular immunity we observed lymphopenia with decreased CD3+ T cells and CD4+ T helper cells in GAS-infected children. We also registered a significantly increased production of pro-inflammatory IFN-γ and IL-6 and no changes in levels of TNFα and IL-1β. On the other hand, we found a reduced level of inflammatory IL-17A and significantly increased anti-inflammatory IL-10. This proves a mixed pattern of pro- and anti-inflammatory host immune responses against GAS infection in children compared to healthy controls, in the setting of resurgence of GAS tonsillopharyngitis and scarlet fever in Bulgaria during 2023. Moreover, our findings are in correlation with the “immune signature” found by Anderson et al. in a human experimental model of GAS-infected adults [54].

## 5. Conclusions

In this study, although of relatively small scale, several important findings become available. The isolated *S. pyogenes* strains were susceptible to the clinically used antibiotics. We observed changes in humoral and cellular immune responses in GAS-infected children. The genetic characteristics of the involved GAS strains revealed a wide variety of virulence factors with the prevalence of the Streptococcal pyrogenic exotoxin SpeA (45%). For the first time, the presence of M1_UK_ lineage was discovered in Bulgaria. The detection of the M1_UK_ lineage and the novel ST (d90b) in Bulgaria, along with the identification of key virulence factors and immune response patterns, highlights the potential for increased virulence and adaptability of *S. pyogenes* strains. These findings call for further research into the epidemiology, genetic evolution, and clinical impact of GAS strains, as well as the implementation of robust monitoring systems to detect emerging lineages and adapt public health strategies accordingly.

## Figures and Tables

**Table 1:** PCR primers and conditions used to differentiate M1_global_ and M1_UK_ *Streptococcus pyogenes* detected by allele-specific PCR.

Target Gene	Primer	Sequences 5′-3′	Product
pstB	Forward-SNP * Forward-WT * Reverse	GATAAATCAATCTTAGATAA GATAAATCAATCTTAGATCA CGTGAGGCTTGCTGCATTGAG	287 bp
gldA	Forward-SNP Forward-WT Reverse	AGATGGGTTAGCAACAAAG AGATGGGTTAGCAACAAGG GAATAGCACCTGTCAGCG	292 bp

* SNP, single-nucleotide polymorphism; WT, wild-type. Forward primers were specifically designed to distinguish between the targeted SNP present in the M1_UK_ lineage and the wild-type (WT) sequence found in the global M1 population.

**Table 2:** Minimal inhibitory concentration (in mg/L) of five antimicrobial agents to 30 *S. pyogenes* strains, isolated during the GAS resurgence in Sofia, 1 January 2023–30 June 2023.

	Erythromycin	Levofloxacin	Moxifloxacin	Tetracycline	Co-Trimoxazole
MIC range	0.023–0.064	0.38–2	0.094–0.5	0.047–0.38	0.002–0.32
MIC_50_	0.036	0.83	0.17	0.12	0.05
MIC_90_	0.054	1.75	0.45	0.32	0.12

MIC—Minimal inhibitory concentration; MIC_50_—MIC of 50% of the tested strains; MIC_90_—MIC of 90% of the tested strains.

**Table 3:** Presentation of the variety of superantigens in the studied *S. pyogenes* clinical strains and their distribution.

Exotoxin Virulence Factors	Produced by Number (%) of Strains
SpeA + SpeJ, including:	18 (45%)
SpeA + SpeJ + sdaD	17
SpeA + SpeJ + sdaD + spd3	11
SpeC, including:	11 (27.5%)
SpeC + SpeI + SpeH + sdaD	1
SpeC + spd3	5
SpeI + SpeH, including:	11 (27.5%)
SpeI + SpeH + sdaD	6
SpeI + sdaD	1

**Table 4:** Single nucleotide polymorphisms (SNPs) found in sample *S. pyogenes* 706, compared to M1_UK_ SNPs, described by Lynskey N. et al., 2019 [42].

	Position in MGAS5005	Gene Locus	Gene	Product	SNP Found in This Study (*S. pyogenes* 706)	Ref.	SNP Found in Lynskey et al. [42]
1	115646	M5005_Spy0106	rofA	Transcriptional regulator	T	C	T
2	116162	M5005_Spy0106	rofA	Transcriptional regulator	C	A	C
3	116163	M5005_Spy0106	rofA	Transcriptional regulator	A	C	A
4	250832	M5005_Spy0243		ABC transporter-associated protein	C	T	C
5	513254	M5005_Spy0525		galactose-6-phosphate isomerase LacB subunit	T	G	T
6	528360	Intergenic		-	T	A	T
7	563631	M5005_Spy0566	sagE	streptolysin S putative self-immunity protein	A	G	A
8	613633	M5005_Spy0609		phosphoglycerol transferase	C	T	C
9	626494	M5005_Spy0623		methyltransferase	A	G	A
10	661707	M5005_Spy0656	trmD	tRNA (guanine-N(1)-)-methyltransferase	A	G	A
11	730823	M5005_Spy0727	recJ	single-stranded-DNA-specific exonuclease	T	C	T
12	784467	M5005_Spy0779		putative membrane spanning protein	C	T	C
13	819098	M5005_Spy0825	murB	UDP-N-acetylenolpyruvoylglucosamine reductase	A	G	A
14	923079	M5005_Spy0933		putative NADH-dependent flavin oxidoreductase	A	G	A
15	942633	M5005_Spy0951	pstB	phosphate transport ATP-binding protein	T	G	T
16	983438	Intergenic		-	C	G	C
17	1082253	M5005_Spy1108	metK2	S-adenosylmethionine synthetase	T	C	T
18	1238124	M5005_Spy1282	msrA	peptide methionine sulfoxide reductase	A	G	A
19	1238673	M5005_Spy1283	tlpA	thiol:disulfide interchange protein	A	G	A
20	1251193	M5005_Spy1293		hypothetical protein	A	G	A
21	1373176	M5005_Spy1400		PTS system, galactose-specific IIB component	A	C	A
22	1407497	M5005_Spy1439		portal protein	T	C	T
23	1446116	M5005_Spy1490		3-oxoacyl-[acyl-carrier protein] reductase	T	C	T
24	1535209	Intergenic		-	G	A	G
25	1702540	M5005_Spy1714	gldA	glycerol dehydrogenase	T	C	T
26	1734749	M5005_Spy1772		glutamate formimidoyltransferase	A	G	A
27	1828734	M5005_Spy1860		putative membrane spanning protein	A	G	A

**Table 5:** Cellular subsets and cytokines in children with GAS infection compared to healthy individuals.

Immune Parameters	Control Group	Patients	*p*
LYMPHOCYTES SUBSETS	means ± SD		
WBC × 10^9^	7.8 ± 2.6	11.9 ± 6.4	0.063
% Lymphocytes	47.7 ± 11.7	28.6 ± 16.56	0.003
% CD3^+^	71 ± 4.7	65 ± 6.4	0.017
% CD4^+^	42.3 ± 6.2	33 ± 7.3	0.004
% CD8^+^	22 ± 3.6	22 ± 5.2	0.60
CD4:CD8 ratio	1.9 ± 0.55	1.58 ± 0.62	0.089
% CD19^+^	16.3 ± 2.22	18.2 ± 5	0.23
%CD3^−^ CD16^+^ 56^+^	11.5 ± 5.05	15 ± 5.1	0.078
CYTOKINES	median (min–max), pg/mL		
IFN-γ	2.73 (2–8)	5.26 (2–75)	0.02
IL-6	1.87 (1–3)	5.83 (1.4–163)	0.000
TNFα	7.88 (6–31)	7.07 (5–33)	0.41
IL-10	3.96 (2–8)	13.05 (2–104)	0.003
IL-17A	25.45 (17–30)	9.5 (15–34)	0.020
IL-1β	7.14 (2–23)	6.4 (3–58)	0.562

## Data Availability

The data presented in this study are included in the article. Further inquiries can be directed to the corresponding author.

## Supplementary Materials

The following Supporting Information can be downloaded at https://www.mdpi.com/article/10.3390/microorganisms13010179/s1, Table S1: PCR Primer Sequences for Detection of *Streptococcus pyogenes* virulence factors by multiplex PCR; Table S2: WGS. DNBSEQ Statistics; Table S3: WGS. The alignment result of *Streptococcus pyogenes*. NCTC12064; Table S4: WGS. The depth and coverage of *Streptococcus pyogenes*. NCTC12064; Table S5: Multi-Locus Sequence Typing (MLST)—PubMLST data; Table S6: Non-synonymous variants detected in *mut*S gene; Table S7: Distribution of *emm* types identified; Table S8: Epidemiology evaluation of the two children infected by *S. pyogenes* M1_UK_.
