# Supplementary material for: Characterization of Streptococcus pyogenes Strains from Tonsillopharyngitis and Scarlet Fever Resurgence, 2023—FIRST Detection of M1UK in Bulgaria
Source: Microorganisms. 2025 Jan 16;13(1):179. doi: 10.3390/microorganisms13010179 (PMC11767604; doi:10.3390/microorganisms13010179)
Supplement: Supplementary file 1 [file microorganisms-13-00179-s001.zip › microorganisms-3409491-supplementary.pdf]

Supplementary 1

Table S1. PCR Primers Sequences for Detection of *Streptococcus pyogenes* virulence factors by multiplex PCR

Mix 1

Target gene	Superantigen	Sequences 5′-3′	Product
speL F	speL Superantigen (streptococcal pyrogenic exotoxin L)	CCTGAGCCGTGAAATTCCCA	657bp
speL R	speL Superantigen (streptococcal pyrogenic exotoxin L)	ACACCAGAATTGTCGTTTGGT	
speK F	speK Superantigen (streptococcal pyrogenic exotoxin K)	CCTTGTGTGTGTATCGCTTGC	568bp
speK R	speK Superantigen (streptococcal pyrogenic exotoxin K)	TTGCTGTCCCCCATCAAACT	
speM F	speM Superantigen (streptococcal pyrogenic exotoxin M)	ATCGCTCATCAAACTTTTCCT	496bp
speM R	speM Superantigen (streptococcal pyrogenic exotoxin M)	CCTTGTGTGTGTATCGCTTGC	
speC F	speC Superantigen (streptococcal pyrogenic exotoxin C)	GCCAATTTCGATTCTGCCGC	405bp
speC R	speC Superantigen (streptococcal pyrogenic exotoxin C)	TGCAGGGTAAATTTTTCAACGACA	
speI F	speI Superantigen (streptococcal pyrogenic exotoxin I)	TTCATAGACGGCGTTCAACAA	176bp
speI R	speI Superantigen (streptococcal pyrogenic exotoxin I)	TGAAATCTAGAGGAGCGGCCA	

Mix 2

Ssa F	Ssa Streptococcal superantigen	AAAGAATACTCGTTGTAGCATGTGT	678bp
Ssa R	Ssa Streptococcal superantigen	AATATTGCTCCAGGTGCGGG	
speA F	speA Superantigen(streptococcal pyrogenic exotoxin A)	AGGTAGACTTCAATTTGGCTTGTGT	576bp
speA R	speA Superantigen(streptococcal pyrogenic exotoxin A)	GGGTGACCCTGTTACTCACG	
speH F	speH Superantigen (streptococcal pyrogenic exotoxin H)	TGAGATATAATTGTCGCTACTCACAT	480bp

speH R	speH Superantigen (streptococcal pyrogenic exotoxin H)	CCTGAGCGGTTACTTTCGGT	
speJ F	speJ Superantigen (streptococcal pyrogenic exotoxin J)	TCCTTGTACTAGATGAGGTTGCAT	286bp
speJ R	speJ Superantigen (streptococcal pyrogenic exotoxin J)	GGTGGGGTTACACCATCAGT	

Mix 3

spd3 F	spd3 Nuclease (Streptodornase)	ATCGTCGTACTTGGCAAGGTT	784bp
spd3 R	spd3 Nuclease (Streptodornase)	GCCGCTTCTTCAAACTCTTCG	
Sdc F	Sdc Nuclease	AAGCTTAGAAACTCTCTCGCCA	600bp
Sdc R	Sdc Nuclease	AGTTCCAGTAATAGCGTTTTTCCGT	
sdaD F	sdaD Nuclease	TTTACGCTGAATCGGGCACT	295bp
sdaD R	sdaD Nuclease	GGCTCTGGTTTGCTTTCCCA	

Table S2. WGS. DNBSEQ Statistics

Sample Name (#)	Insert Size (bp)	Reads Length (bp)	Raw Data (Mb)	Adapter (%)	Duplication (%)	Total Reads (#)	Filtered Reads (%)	Low Quality Filtered Reads (%)	Clean Data (Mb)
Str266	350	(150:150)	1,310	3.63	3.14	8,738,130	6.96	0	1,219
Str279	350	(150:150)	1,310	2.78	2.94	8,738,130	5.91	0	1,233
Str296	350	(150:150)	1,310	2.00	3.11	8,738,130	5.30	0	1,241
Str582	350	(150:150)	1,310	2.38	1.73	8,738,130	4.46	0	1,252
Str706	350	(150:150)	1,310	2.33	1.68	8,738,130	4.34	0	1,253
Str799	350	(150:150)	1,310	3.57	1	8,738,130	4.66	0	1,249
Str826	350	(150:150)	1,310	1.61	1.87	8,738,130	3.66	0	1,262

Table S3. WGS. The alignment result of *Streptococcus pyogenes*. NCTC12064

Sample_Name	Clean reads	Clean bases(bp)	Mapping rate	Unique rate	Duplicate rate	Mismatch rate
Str266	1,164,256	174,638,400	85.75	97.78	0.00	0.89
Str279	1,164,256	174,638,400	86.18	98.02	0.00	0.87
Str296	1,164,256	174,638,400	84.72	98.12	0.00	0.91
Str582	1,164,256	174,638,400	81.86	96.89	0.00	0.90
Str706	1,164,256	174,638,400	85.22	97.03	0.00	0.93
Str799	1,164,256	174,638,400	83.59	97.33	0.00	0.91
Str826	1,164,256	174,638,400	85.21	97.42	0.00	0.94

Table S4. WGS. The depth and coverage of *Streptococcus pyogenes*. NCTC12064

Sample	Referance Name	Referance Size(bp)	Coverage	Average sequencing depth(X)
Str266	Streptococcus.pyogenes.NCTC12064	1,746,380	93.40%	84.66
Str279	Streptococcus.pyogenes.NCTC12064	1,746,380	95.35%	85.14
Str296	Streptococcus.pyogenes.NCTC12064	1,746,380	93.98%	83.64
Str582	Streptococcus.pyogenes.NCTC12064	1,746,380	93.88%	80.26
Str706	Streptococcus.pyogenes.NCTC12064	1,746,380	94.45%	83.58
Str799	Streptococcus.pyogenes.NCTC12064	1,746,380	94.02%	82.54
Str826	Streptococcus.pyogenes.NCTC12064	1,746,380	94.01%	84.28

Table S5. Multi-Locus Sequence Typing (MLST) - PubMLST data:

gki	gtr	murI	mutS	recP	xpt	yqiL
STR266.fasta spyogenes - gki(5) gtr(2) murI(2) mutS(~6) recP(6) xpt(2) yqiL(2)						
5	2	2	6 New (d90b)	6	2	2
STR279.fasta spyogenes 101 gki(16) gtr(2) murI(8) mutS(3) recP(1) xpt(13) yqiL(3)						
16	2	8	3	1	13	3
STR296.fasta spyogenes - gki(5) gtr(2) murI(2) mutS(~6) recP(6) xpt(2) yqiL(2)						
5	2	2	6 New (d90b)	6	2	2
STR582.fasta spyogenes 242 gki(5) gtr(2) murI(2) mutS(82) recP(6) xpt(2) yqiL(2)						
5	2	2	82	6	2	2
STR706.fasta spyogenes 28 gki(4) gtr(3) murI(4) mutS(4) recP(4) xpt(2) yqiL(4)						
4	3	4	4	4	2	4
STR799.fasta spyogenes - gki(5) gtr(2) murI(2) mutS(~6) recP(6) xpt(2) yqiL(2)						
5	2	2	6 New (d90b)	6	2	2
STR826.fasta spyogenes - gki(5) gtr(2) murI(2) mutS(~6) recP(6) xpt(2) yqiL(2)						
5	2	2	6 New (d90b)	6	2	2

Table S6. Non-synonymous variants detected in *mut*S gene:
Str266, Str296, Str799 and Str826 show a pattern of 10 SNPs expected to result in amino acid substitutions in MutS protein. The novel haplotype (d90b) is highlighted in yellow.

	ref	Str266	Str279	Str296	Str582	Str706	Str799	Str826	Ref base<->sample base	cdsPos: phase	proPos	Ref codon<->sample codon	Ref aa<->sample aa	Mutate type	ref_gene_ID
NZ_LS483338.1: 1681874	G	A	G	A	A	G	A	A	G<->A	2512	838	CTT<->TTT	L<->F	nonsyn	DQM35_RS08575
NZ_LS483338.1: 1681928	T	C	T	C	C	C	C	C	T<->C	2458	820	ACT<->GCT	T<->A	nonsyn	DQM35_RS08575
NZ_LS483338.1: 1681935	T	A	T	A	A	T	A	A	T<->A	2451	817	GAA<->GAT	E<->D	nonsyn	DQM35_RS08575
NZ_LS483338.1: 1681994	A	G	A	G	G	A	G	G	A<->G	2392	798	TCA<->CCA	S<->P	nonsyn	DQM35_RS08575
NZ_LS483338.1: 1682004	T	C	T	C	C	T	C	C	T<->C	2382	794	ATA<->ATG	I<->M	nonsyn	DQM35_RS08575
NZ_LS483338.1: 1682208	C	C	C	C	C	G	C	C	C<->G	2178	726	AAG<->AAC	K<->N	nonsyn	DQM35_RS08575
NZ_LS483338.1: 1682516	T	T	T	T	G	T	T	T	T<->G	1870	624	ATG<->CTG	M<->L	nonsyn	DQM35_RS08575
NZ_LS483338.1: 1682744	T	C	T	C	C	C	C	C	T<->C	1642	548	ATT<->GTT	I<->V	nonsyn	DQM35_RS08575
NZ_LS483338.1: 1682989	G	T	G	T	T	T	T	T	G<->T	1397	466	ACT<->AAT	T<->N	nonsyn	DQM35_RS08575
NZ_LS483338.1: 1683269	T	C	T	C	C	C	C	C	T<->C	1117	373	AAC<->GAC	N<->D	nonsyn	DQM35_RS08575
NZ_LS483338.1: 1683317	G	A	G	A	A	G	A	A	G<->A	1069	357	CAT<->TAT	H<->Y	nonsyn	DQM35_RS08575
NZ_LS483338.1: 1684382	C	T	C	T	T	C	T	T	C<->T	4	2	GCA<->ACA	A<->T	nonsyn	DQM35_RS08575

Table S7. Distribution of *emm* types identified:

266	emm	12	180bp
	emmPrime	2	19bp
	mga-1	5	36bp
	SF1	1	26bp
279	emm	89	180bp
	emmL	203.4	180bp
	emmPrime	2	19bp
	mga-2	9	32bp
	SF2	1	30bp
	SF3	1	30bp
	SF4	9	54bp
296	emm	12	180bp
	emmPrime	2	19bp
	mga-1	5	36bp
	SF1	1	26bp

582	emm	12.101	180bp
	emmPrime	2	19bp
	SF1	1	26bp
706	emm	1	180bp
	emmPrime	2	19bp
	mga-1	5	36bp
	SF1	1	26bp
799	emm	12	180bp
	emmPrime	2	19bp
	mga-1	5	36bp
	SF1	1	26bp
826	emm	12	180bp
	emmPrime	2	19bp
	mga-1	5	36bp
	SF1	1	26bp

Table S8. Epidemiology evaluation of the two children infected by *S. pyogenes* $M1_{UK}$

	Age (year)	Sex	Diagnosis	Attendance in	Residence	$M1_{UK}$ positive throat swab (date)	PCR-detected virulence factors	History of contact with a *sick person in the previous 5 days*	History of travelling abroad and/or contact with a person who travelled abroad in the last month prior to infection
Case 1	6	female	tonsillo-pharyngitis	kinder-garten	Sofia	13 March 2023	SpeA+SpeJ+ sdaD+pstB	Yes	No
Case 2	7	male	tonsillo-pharyngitis	elementary school	Sofia	24 June 2023	SpeA+SpeJ+ sdaD+pstB	Yes	No
